# Functional characterization and clinical significance of IGSF8 in pan-cancer: an integrated bioinformatic and experimental study

**DOI:** 10.3389/fimmu.2025.1642193

**Published:** 2025-08-27

**Authors:** Jie Wang, Lingxiao Lu, Ruicheng Wu, Dengxiong Li, Zhipeng Wang, Luxia Ye, Dechao Feng

**Affiliations:** ^1^ Department of Urology, The First Affiliated Hospital of Zhejiang Chinese Medical University (Zhejiang Provincial Hospital of Chinese Medicine), Hangzhou, Zhejiang, China; ^2^ Department of Urology, Institute of Urology, West China Hospital, Sichuan University, Chengdu, China; ^3^ Department of Public Research Platform, Taizhou Hospital of Zhejiang Province Affiliated to Wenzhou Medical University, Linhai, China; ^4^ Department of Urology, Sichuan Provincial People’s Hospital, University of Electronic Science and Technology of China, Chengdu, China; ^5^ Division of Surgery and Interventional Science, University College London, London, United Kingdom

**Keywords:** immunoglobulin superfamily member 8, pan-cancer analysis, tumor immune microenvironment, drug sensitivity, tumor biomarker

## Abstract

**Background:**

Immunoglobulin superfamily member 8 (IGSF8) is a membrane protein implicated in crucial biological processes like cell interactions and immune responses. Emerging evidence suggests that IGSF8 plays a significant role in various cancers by influencing tumor progression through regulation of cell proliferation, migration, and apoptosis. Analyzing its expression, mutation status, and clinical correlations across different cancer types through pan-cancer bioinformatics could provide valuable insights into its potential as a biomarker and target for cancer therapies.

**Methods:**

In this study, we utilized several public databases to investigate the biological role of IGSF8, focusing on its associations with prognosis, tumor heterogeneity, stemness, immune checkpoint genes, and immune cell infiltration across different types of cancer. Additionally, the GDSC and CTRP databases were employed to assess the sensitivity of IGSF8 to small molecule drugs. CCK8 assay and colony formation assay were used to detect its biological effect on cancer cells.

**Results:**

IGSF8 was significantly upregulated in 23 types of cancers and associated with poor prognosis in several cancers, including cell carcinoma and endocervical adenocarcinoma (CESC) and Acute Myeloid Leukemia(LAML). Its high expression was linked to multiple immune regulatory genes and immune checkpoint genes in the tumor microenvironment, with a notable positive correlation with CD276 in most cancers. IGSF8 was also closely associated with multiple indicators of tumor heterogeneity, stemness, as well as significant RNA methylation modifications across various cancers. Drug sensitivity analysis identified BX-795 and tozasertib as potential treatments for tumors with high IGSF8 expression. Knockdown of IGSF8 significantly inhibited the proliferation ability of prostate cancer cells.

**Conclusion:**

Our findings indicated that IGSF8 might be used as a potential prognostic marker and therapeutic target for various cancers.

## Introduction

The global burden of malignant neoplasms manifests as a substantial public health challenge, characterized by escalating incidence rates, significant mortality, disability-adjusted life years and profound socioeconomic costs across healthcare systems worldwide (1, 2). Cancer treatment has advanced significantly over the past century, shifting from early methods like surgery and radiation to chemotherapy, targeted therapies, and immunotherapies (3–5). This evolution reflects a move from non-specific cytotoxic approaches to precision strategies guided by molecular and immune profiling. Currently, two major treatment paradigms are widely applied. One targets cancer cells directly through cytotoxic or molecular agents, offering rapid tumor shrinkage but often limited by drug resistance and adverse effects (6, 7). The other modulates the tumor microenvironment to restore immune surveillance and block tumor-promoting pathways, potentially offering more sustained benefits but requiring individualized adaptation (8). New therapeutic strategies are emerging that aim to combine these approaches. Innovations such as bispecific antibodies, oncolytic viruses, and personalized vaccines integrate direct tumor targeting with immune activation, offering promising directions for future cancer care (9–11).

Immunoglobulin superfamily member 8 (IGSF8), also known as CD316 or EWI-2, is a type I transmembrane protein that belongs to the immunoglobulin superfamily (12). It was initially characterized as a binding partner of tetraspanins CD9 and CD81, modulating the organization and vesicular trafficking of tetraspanin-enriched membrane domains (TEMDs) (13, 14). Through these interactions, IGSF8 influences the activity of growth factor receptors, cell adhesion proteins, and their downstream signaling cascades (15, 16). Beyond membrane organization, IGSF8 is also involved in neuronal migration, axon guidance, and synapse formation (17), suggesting its broad regulatory roles across multiple biological systems. Recent studies have linked IGSF8 dysregulation to the pathogenesis of various diseases, including neurological disorders and cancers (18). Intriguingly, IGSF8 exhibits dual roles in tumor biology, acting either as a tumor suppressor or a tumor promoter depending on the cellular context. In certain solid tumors such as melanoma, lung, prostate cancer, and glioma, IGSF8 was shown to suppress tumor progression by modulating pathways like TGF-β and EGFR-MAPK, thereby inhibiting proliferation and metastasis (19–22). In contrast, in hematological malignancies such as acute myeloid leukemia, IGSF8 sustains leukemic stemness by stabilizing β-catenin, preventing its degradation and enhancing Wnt pathway activation, which in turn promotes therapy resistance and disease progression (12). These contrasting roles suggest that IGSF8 exerts context-specific effects through distinct signaling mechanisms. Moreover, IGSF8 has emerged as an innate immune checkpoint molecule in the tumor microenvironment (TME). It suppresses NK cell-mediated cytotoxicity by interacting with immune inhibitory receptors, contributing to immune evasion particularly in “cold” tumors that are poorly infiltrated by cytotoxic immune cells (23). Blocking this interaction enhances NK cell killing capacity and promotes immune activation, offering a promising therapeutic avenue to convert immunologically “cold” tumors into “hot” ones responsive to immunotherapy. Therefore, the impact of IGSF8 on prognosis may not only reflect its intrinsic roles in tumor signaling and stemness, but also its influence on shaping immune landscapes across different cancer types.

Despite these emerging insights, a comprehensive pan-cancer evaluation of IGSF8 remains lacking. Identification of tumor biomarkers serves as the cornerstone of precision oncology, enabling the implementation of tailored therapeutic strategies that significantly enhance treatment efficacy and improve survival outcomes (24–26). To fill this knowledge gap, we conducted a systematic investigation using The Cancer Genome Atlas (TCGA) data to examine the expression, prognostic relevance, immune associations, and therapeutic implications of IGSF8 across a wide range of human cancers. Our findings reveal the multifaceted roles of IGSF8 and support its potential as a prognostic biomarker and therapeutic target in precision oncology.

## Materials and methods

### Date acquisition, IGSF8 expression and survival analysis

The subcellular localization and structure of the protein encoded by IGSF8 were studied using the Human Protein Atlas database (https://www.proteinatlas.org). The gene-gene interaction network of IGSF8 was obtained from the Genemania database (http://genemania.org/) (27). We obtained the TCGA pan-cancer dataset from the USCS database and our previous study (28–31). IGSF8 expression data were obtained from the TCGA prognostic dataset, excluding samples with an expression level of 0, and covering a range of sample types, including normal solid tissues, primary tumors, and hematologic cancers. The correlation between IGSF8 expression and clinical parameters, such as tumor stage and grade, was explored (29). Patients were divided into high and low expression groups according to the median IGSF8 expression value. Furthermore, high-quality prognostic datasets derived from previous TCGA studies were incorporated (28). Cox proportional hazards regression analysis was applied to assess the prognostic impact of IGSF8, with overall survival (OS), disease-specific survival (DSS), disease-free survival (DFS), and progression-free interval (PFI) serving as key endpoints (29, 32).

### Tumor heterogeneity, stemness and gene mutation analysis

Spearman correlation analysis was conducted to evaluate the relationships among tumor heterogeneity indicators, including tumor mutation burden (TMB), tumor purity, neoantigen (NEO), microsatellite instability (MSI), and IGSF8 expression levels (29, 33). Additionally, Spearman analysis was applied to assess the correlation between tumor stemness features, such as DNA methylation score (DNAss) and RNA expression score (RNAss) (29, 34), and IGSF8 expression. For gene mutation analysis, the Mutect2 software was used to process a simple nucleotide variation dataset, allowing for the identification of gene mutations. After integrating the data, gene expression and mutations were analyzed in cancer types such as Colon adenocarcinoma (COAD), Brain Lower Grade Glioma (LGG), Liver hepatocellular carcinoma (LIHC), Lung squamous cell carcinoma (LUSC), and Stomach adenocarcinoma (STAD) (29). To assess differences in mutation frequency across sample groups, a chi-square test was performed.

### Tumor immune microenvironment, RNA modifications and drug sensitivity

In our study of the tumor immune microenvironment, we assessed the correlation between IGSF8 mRNA expression and a comprehensive set of immune-related factors. This included 36 stimulatory and 24 heterogeneous immune checkpoints, along with 150 immune regulatory genes, encompassing receptors, MHC molecules, chemokines, and both immunoinhibitory and immunostimulatory factors (29, 35). Tumor-infiltrating cells were evaluated using the EPIC, CIBERSORT, MCPcounter, Timer and xCELL algorithm to determine their composition and distribution within the TME (29, 36). Additionally, we investigated the relationship between IGSF8 and RNA methylation regulators, focusing on m1A, m6A, and m5C modifications (29). Furthermore, the potential impact of IGSF8 expression on drug sensitivity across various cancer types was analyzed using GSCALite (37), which integrates data from the Genomics of Drug Sensitivity in Cancer (GDSC) and Cancer Treatment Response Portal (CTRP) databases. Positive correlations with drug resistance and negative correlations with drug sensitivity were examined to elucidate IGSF8’s role in modulating therapeutic responses.

### Cell culture

Human prostate cancer cell lines, PC3 and DU145, were purchased and authenticated from cell bank (Chinese Academy of Sciences, Shanghai, China). DU145 and PC3 were cultured in RPMI medium 1640 (Gibco) with 10% FBS and 1% penicillin-streptomycin solution. All cells were incubated in 5% CO2 incubator at 37°C and were tested for mycoplasma free via a mycoplasma detection kit (Thermo Fisher Scientific, United States).

### Real-time quantitative polymerase chain reaction

Total RNA was isolated using TRIzol^®^ reagent (#9109, Thermo Scientific, Japan). Next, the isolated RNA was synthesized into cDNA using PrimeScript™ RT Master Mix reagent (#RR036A, TakaRa, Japan). After that, quantitative PCR (qPCR) was performed according to the manufacturer’s instructions for the TB Green^®^ Premix reagent (#RR820A, TaKaRa, Japan). Glyceraldehyde-3-phosphate dehydrogenase (GAPDH) was used as an internal control. GAPDH: 5’- GTCTCCTCTGACTTCAACAGCG-3’ (forward) and 5’-ACCACCCTGTTGCTGTAGCCAA-3’ (reverse); The primers used for the qPCR assay were IGSF8: 5’- TGCAATGTGACCGGCTATGAG-3’ (forward) and 5’- CCACCACTCGGGACTTGAAG-3’ (reverse). The relative mRNA expression of each detected gene was calculated using the 2^(-ΔΔCt)^ method.

### Cell transfections, cell viability assay and colony forming assay

Prostate cancer cells were transfected using pGLV3 lentiviral vector synthesized by GenePharma Corporation (Shanghai, China). IGSF8 shRNA#1: 5ʹ-GAAGGTGGCATCCAGAACATA-3ʹ; IGSF8 shRNA#2: 5ʹ-CCTTGGAACTGCTGTGCAATG-3ʹ; IGSF8 shRNA#3: 5ʹ-ACTTCGAGTGGTTCCTGTATA-3ʹ. RT-qPCR was used to determine the effective shRNAs of IGSF8. CCK-8 (#C0121, Biosharp, Anhui) was used to assess the viability of prostate cancer cells. According to the reagent instructions, 3×10^3^ prostate cancer cells were cultured in 96-well plates. 10 μl of CCK-8 reagent was added to prostate cancer cells, which were seeded in a 96-well plate in a humidified 5% CO2 atmosphere at 37°C for 1 h. The optical density (OD) was measured with a microplate reader (Thermo Fisher Scientific) at 450 nm. For colony forming assay, a total of 1×10^3^ prostate cancer cells were seeded into 35 mm dishes supplemented with 2 mL of culture medium containing 10% FBS. After 7 days of culture, the cells were fixed, washed with PBS and stained with 0.1% crystal violet solution (#C0121, Beyotime, Beijing, China). The clone clusters in the dishes were scanned and counted by using ImageJ software (version 1.0, NIH,USA).

### Statistical analysis

Unpaired Wilcoxon rank sum and signed rank tests assessed pairwise differences, while the Kruskal test was used for multiple sample sets. For survival analysis, we used the coxph function from the R package survival (version 3.2-7) to build a Cox proportional hazards regression model (32) in order to analyze the relationship between gene expression and prognosis in each type of cancer. Spearman analysis evaluated correlations among continuous variables that failed the Shapiro–Wilk normality test. These analyses were performed using the Sanger platform (29). A p-value less than 0.05 was considered statistically significant, denoted as follows: not significant (ns), P>0.05; *, P< 0.05; **, P<0.01; ***, P<0.001.

## Results

### Basic information and predicted genes interacted with IGSF8

**Figure 1:**
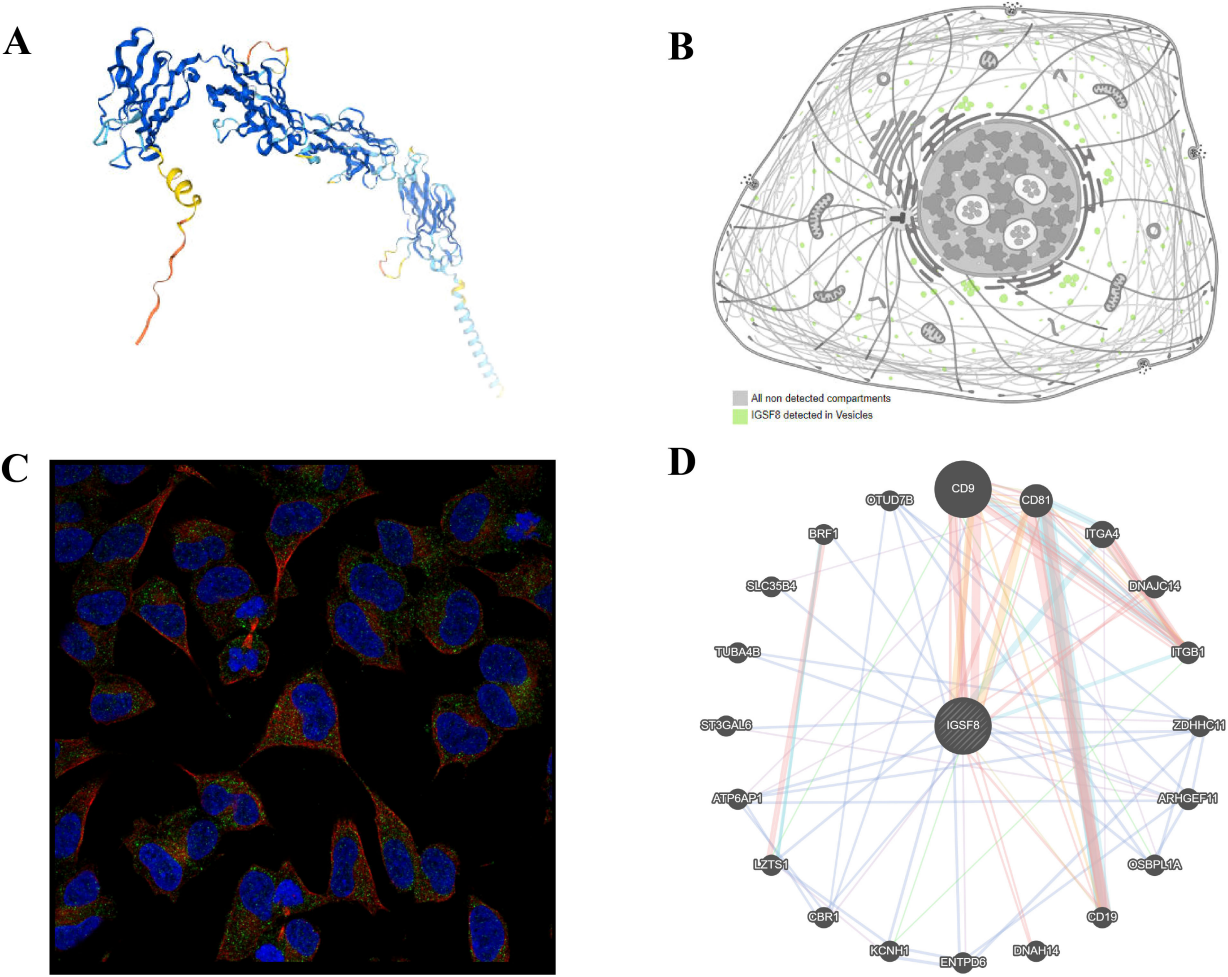
Protein localization of IGSF8. **(A)** the protein structure of IGSF8; **(B)** the subcellular localization of IGSF8; **(C)** the subcellular localization of IGSF8 by immunofluorescence staining; **(D)** protein-protein interaction network of IGSF8.

We obtained the protein structure of IGSF8 (**Figure 1A**) and its localization to the membrane and vesicles from HPA analysis tool (**Figures 1B, C**). The gene–gene interaction network for IGSF8 was constructed by GeneMANIA. The results showed the top 20 predicted genes interacted with IGSF8, in which CD9 ranked the first (**Figure 1D**).

### Differential expression and prognosis analysis of IGSF8

**Figure 2:**
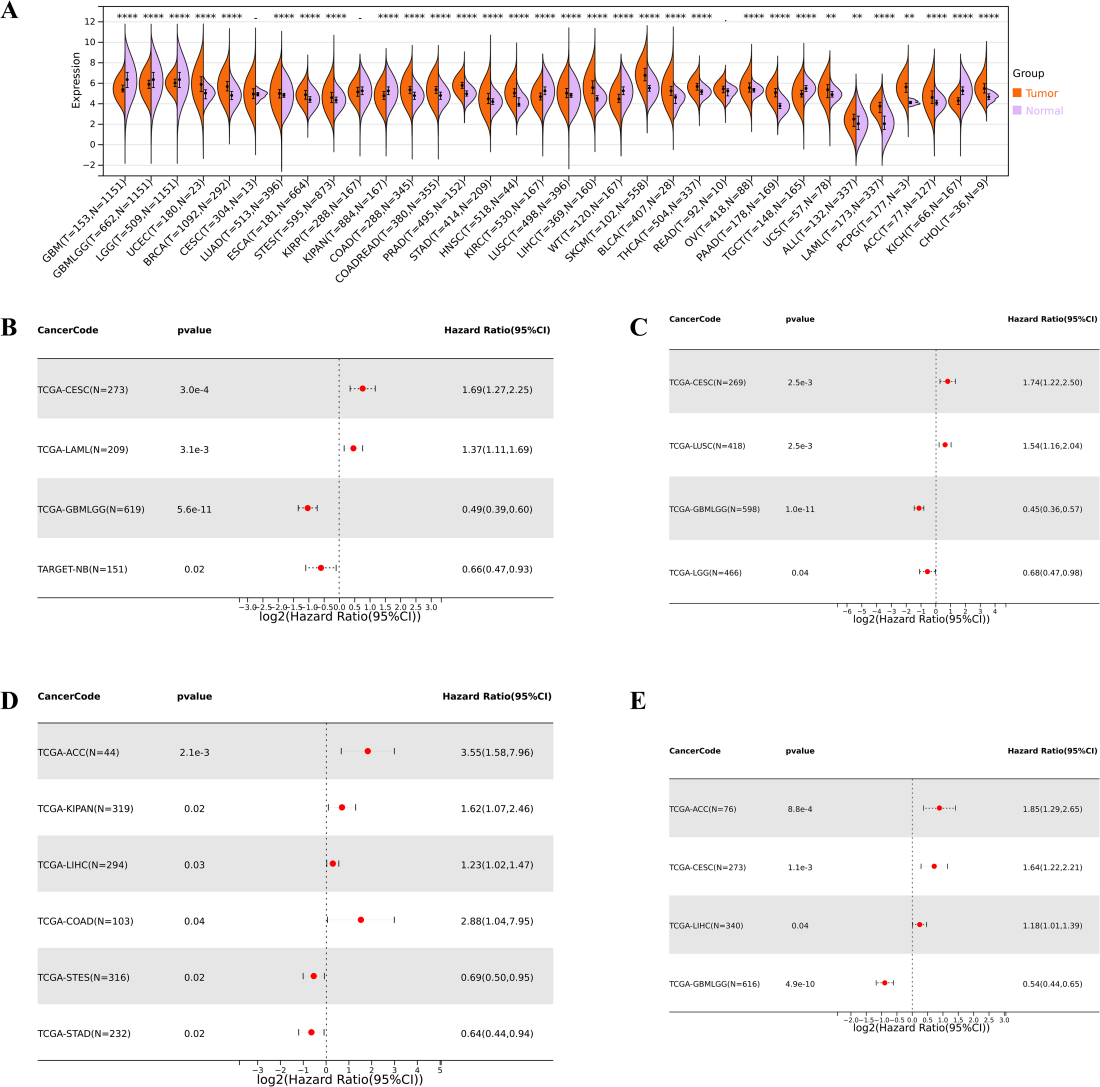
Differential expression and prognosis analyses of IGSF8 at pan-cancer level. **(A)** IGSF8 mRNA expression between tumor and normal tissues at pan-cancer level. **(B)** pan-cancer analysis of IGSF8 for overall survival; **(C)** pan-cancer analysis of IGSF8 for disease-specific survival; **(D)** pan-cancer analysis of IGSF8 for disease free interval; **(E)** pan-cancer analysis of IGSF8 for progression-free interval. *p < 0.05; **p < 0.01; ****p < 0.0001.

Compared to normal samples, we found that the IGSF8 mRNA expression was significantly upregulated in 23 cancers, including Uterine Corpus Endometrial Carcinoma (UCEC), Breast invasive carcinoma (BRCA), Lung adenocarcinoma (LUAD), Esophagus carcinoma (ESCA), Stomach and Esophageal carcinoma (STES), COAD, Colon adenocarcinoma/Rectum adenocarcinoma (COADREAD), Prostate adenocarcinoma (PRAD), STAD, Head and Neck squamous cell carcinoma (HNSC), LUSC, LIHC, Skin Cutaneous Melanoma (SKCM), Bladder Urothelial Carcinoma (BLCA), Thyroid carcinoma (THCA), Ovarian serous cystadenocarcinoma (OV), Pancreatic adenocarcinoma (PAAD), Uterine Carcinosarcoma (UCS), Acute Lymphoblastic Leukemia (ALL), Acute Myeloid Leukemia (LAML), Pheochromocytoma and Paraganglioma (PCPG), Adrenocortical carcinoma (ACC) and Cholangiocarcinoma (CHOL) (**Figure 2A**). In terms of OS, we observed a significant association between high expression of IGSF8 and poor prognosis in several cancer types, including Cervical squamous cell carcinoma and endocervical adenocarcinoma (CESC) and LAML (**Figure 2B**). In terms of DSS, we observed a significant correlation between high expression of IGSF8 and poor prognosis in patients with CESC and LUSC (**Figure 2C**). In terms of DFI, high expression of IGSF8 was associated with poor prognosis in patients with ACC, Pan-kidney cohort (KIPAN), LIHC and COAD (**Figure 2D**). In terms of PFI, high expression of IGSF8 was associated with poor prognosis in patients with ACC, CESC and LIHC (**Figure 2E**). Furthermore, we found that IGSF8 expression levels in STES were associated with T stage, N stage, different clinical stages, grades and genders and IGSF8 expression levels in KIPAN were associated with T stage, N stage, M stage, different clinical stages and grades (**Supplementary Figure S1**).

### Tumor immune microenvironment analysis and drug sensitivity

**Figure 3:**
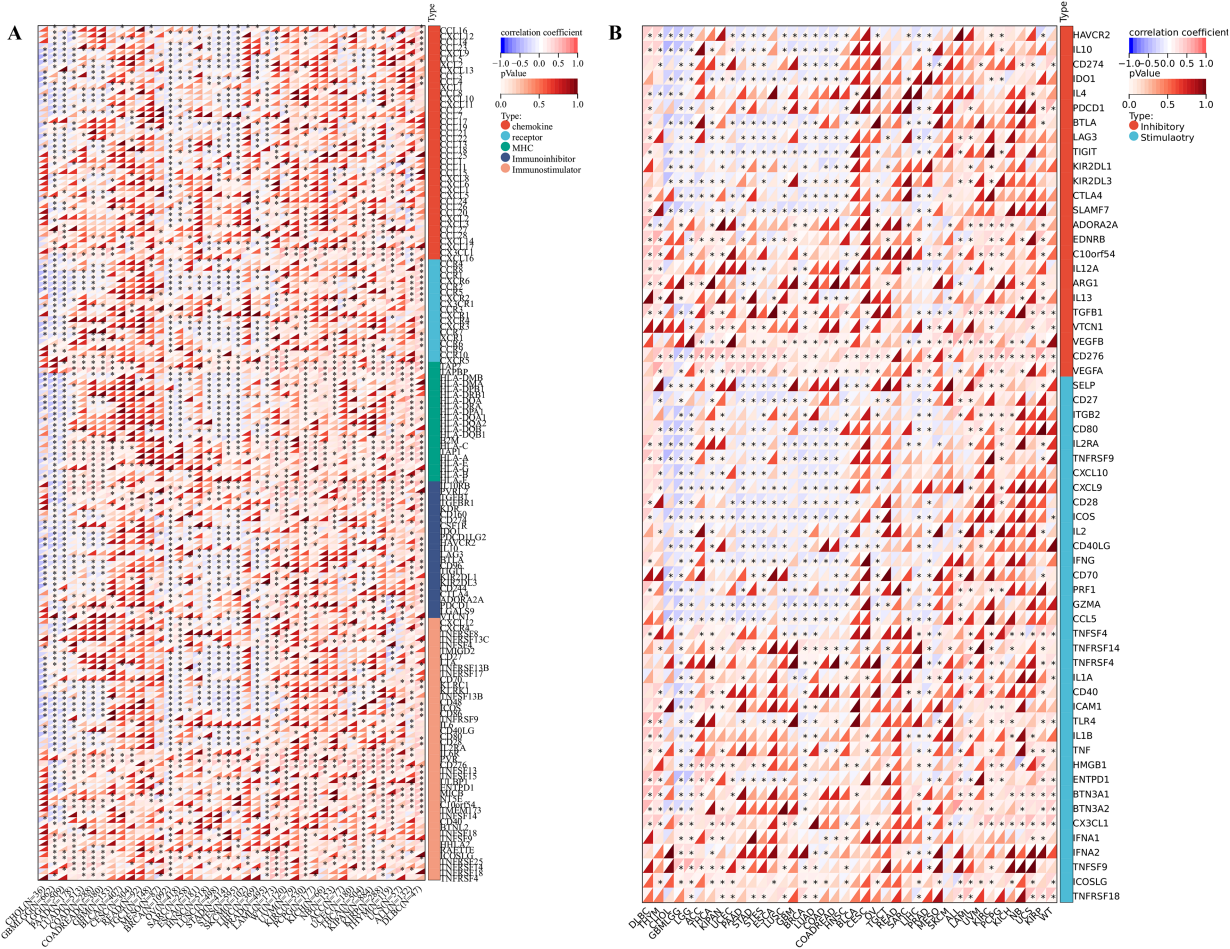
IGSF8 expression with immunoregulatory genes and immune checkpoints. **(A)** the correlation between immunomodulatory genes and IGSF8 at pan-cancer level; **(B)** the correlation between immune checkpoints genes and IGSF8 at pan-cancer level. *p < 0.05.

**Figure 4:**
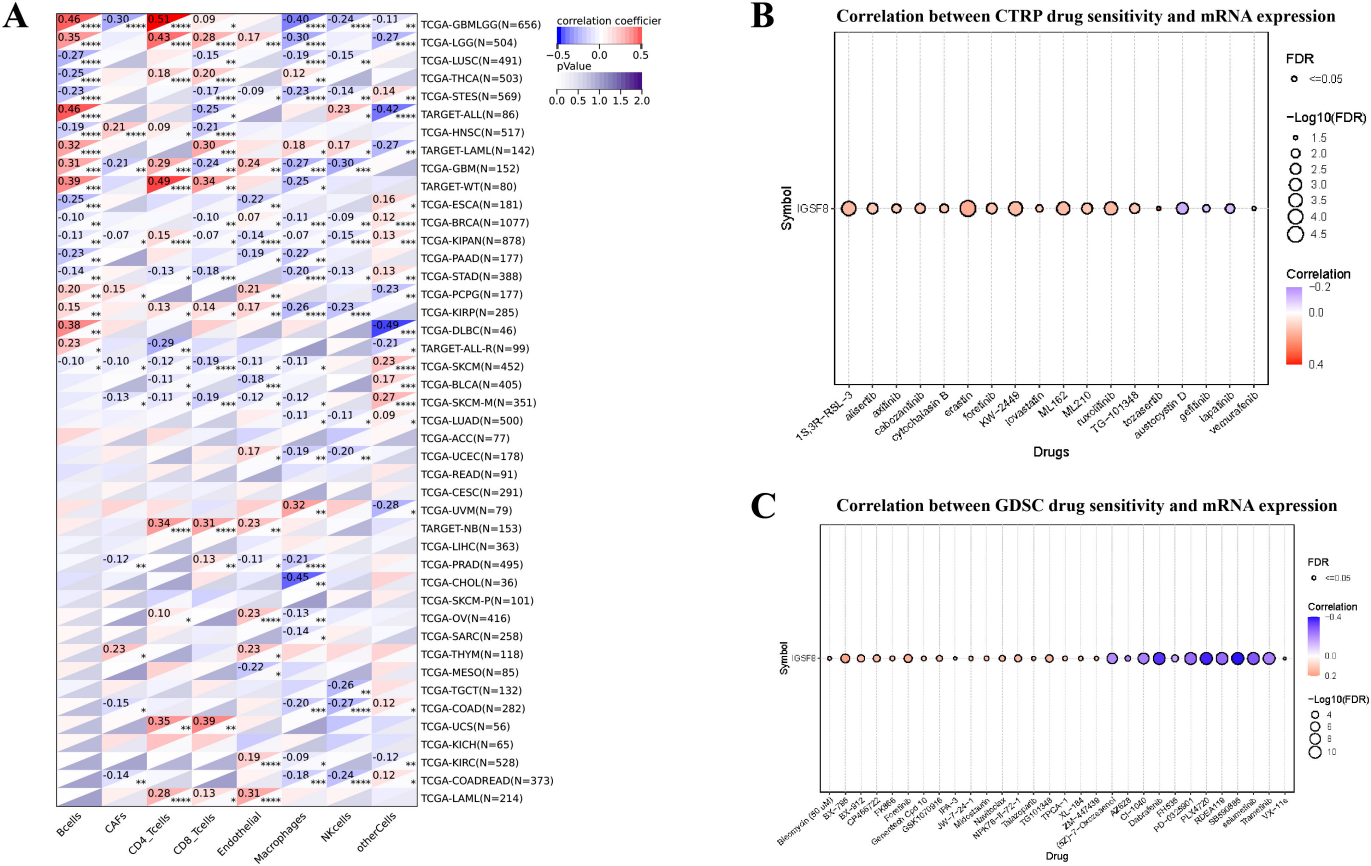
The correlation between IGSF8 expression and tumor-infiltrating cells and drug sensitivity. **(A)** the correlation between tumor-infiltrating cells and IGSF8 at pan-cancer level using the EPIC algorithm; **(B)** the correlation between IGSF8 expression and CTRP drugs sensitivity (top 18) in pan-cancer; **(C)** the correlation between IGSF8 expression and GDSC drugs sensitivity (top 30) in pan-cancer. Positive correlation indicates a higher IGSF8 expression may lead to drug resistance whereas the negative correlation suggests that high IGSF8 expression may lead to drug sensitivity. *p < 0.05; **p < 0.01; ***p < 0.001; ****p < 0.0001.

For the tumor immune microenvironment analysis, our findings indicated that the expression levels of IGSF8 in various cancer types were associated with multiple immune regulatory genes and immune checkpoint genes (**Figures 3A, B**). Specifically, In Glioma (GBMLGG) and BRCA, we observed that IGSF8 expression levels were negatively correlated with most of the immunoregulatory genes (**Figure 3A**). In addition, IGSF8 displayed a remarkable positively correlation with most MHC in KIRC patients (**Figure 3A**). Similarly, IGSF8 was negatively associated with abundant immune checkpoints in GBMLGG (**Figure 3B**). Notably, we observed that IGSF8 expression levels showed a significant positively relationship with CD276 in most malignancies, suggesting the necessity to further explore the mechanisms involved. For tumor-infiltrating lymphocytes, we used EPIC algorithm to evaluate the composition and distribution within the TME. We found in GBMLGG, IGSF8 expression levels were negatively associated with cancer associated fibroblasts (CAFs), macrophages, NK cells and positively associated with B cells and CD4+ T cells (**Figure 4A**). In LGG, IGSF8 expression levels were negatively associated with macrophages and positively associated with B cells, CD4+ T cells, CD8+ T cells and endothelial cells (**Figure 4A**). In TARGET-WT, IGSF8 expression levels were negatively associated with macrophages and positively associated with B cells, CD4+ T cells and CD8+ T cells (**Figure 4A**). We obtained similar results using CIBERSORT, MCPcounter, Timer and xCELL algorithms (**Supplementary Figure S2**). Next, we utilized GDSC and CTRP databases to find drugs targeting tumors with high IGSF8 expression (**Figures 4B, C**). Among the test drugs, the GDSC dataset showed that BX-795 (r=0.18) had the strongest correlation with IGSF8 expression (**Figure 4B**). In addition, the CTRP database indicated tozasertib (r=0.33) had the strongest correlation with IGSF8 expression (**Figure 4C**).

### Tumor heterogeneity, stemness, mutation and RNA methylation analysis

**Figure 5:**
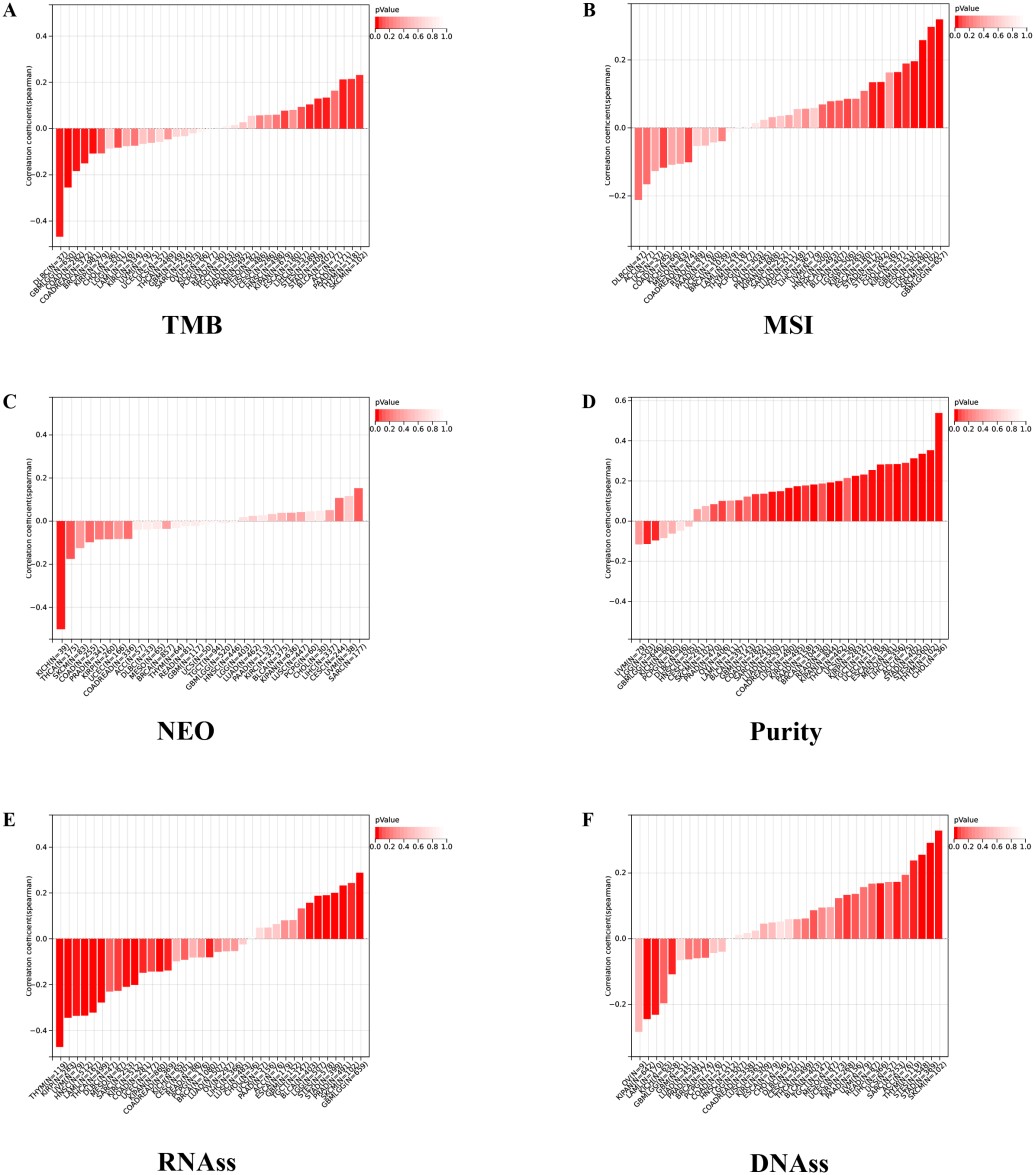
Tumor heterogeneity and stemness analyses. **(A)** the correlation between IGSF8 expression and TMB at pan-cancer level; **(B)** the correlation between IGSF8 expression and MSI at pan-cancer level; **(C)** the correlation between IGSF8 expression and NEO at pan-cancer level; **(D)** the correlation between IGSF8 expression and purity at pan-cancer level; **(E)** the correlation between IGSF8 expression and RNAss at pan-cancer level; **(F)** the correlation between IGSF8 expression and DNAss at pan-cancer level. TMB tumor mutational burden, MSI microsatellite instability, NEO neoantigen, RNAss RNA expression score, DNAss DNA methylation score.

**Figure 6:**
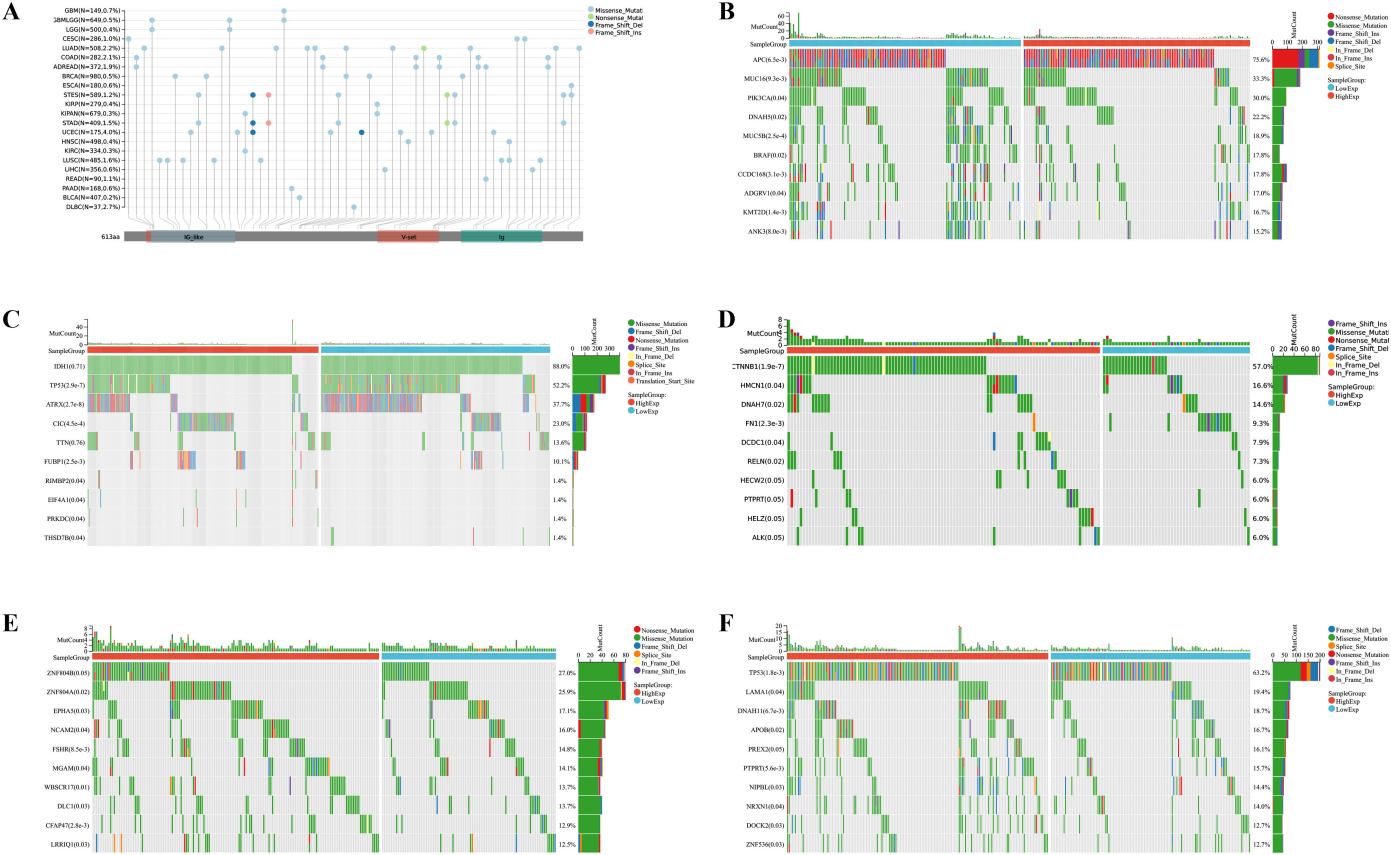
Mutation landscape of IGSF8. **(A)** mutation landscapes of IGSF8 at pan-cancer level; **(B)** the differences in gene mutation frequency between high- and low-IGSF8 expression groups in COAD; **(C)** the differences in gene mutation frequency between high- and low-IGSF8 expression groups in LGG; **(D)** the differences in gene mutation frequency between high- and low-IGSF8 expression groups in LIHC. **(E)** the differences in gene mutation frequency between high- and low-IGSF8 expression groups in LUSC. **(F)** the differences in gene mutation frequency between high- and low-IGSF8 expression groups in STAD. COAD colon adenocarcinoma, LGG lower grade glioma, LIHC liver hepatocellular carcinoma, LUSC lung squamous cell carcinoma, STAD stomach adenocarcinoma.

**Figure 7:**
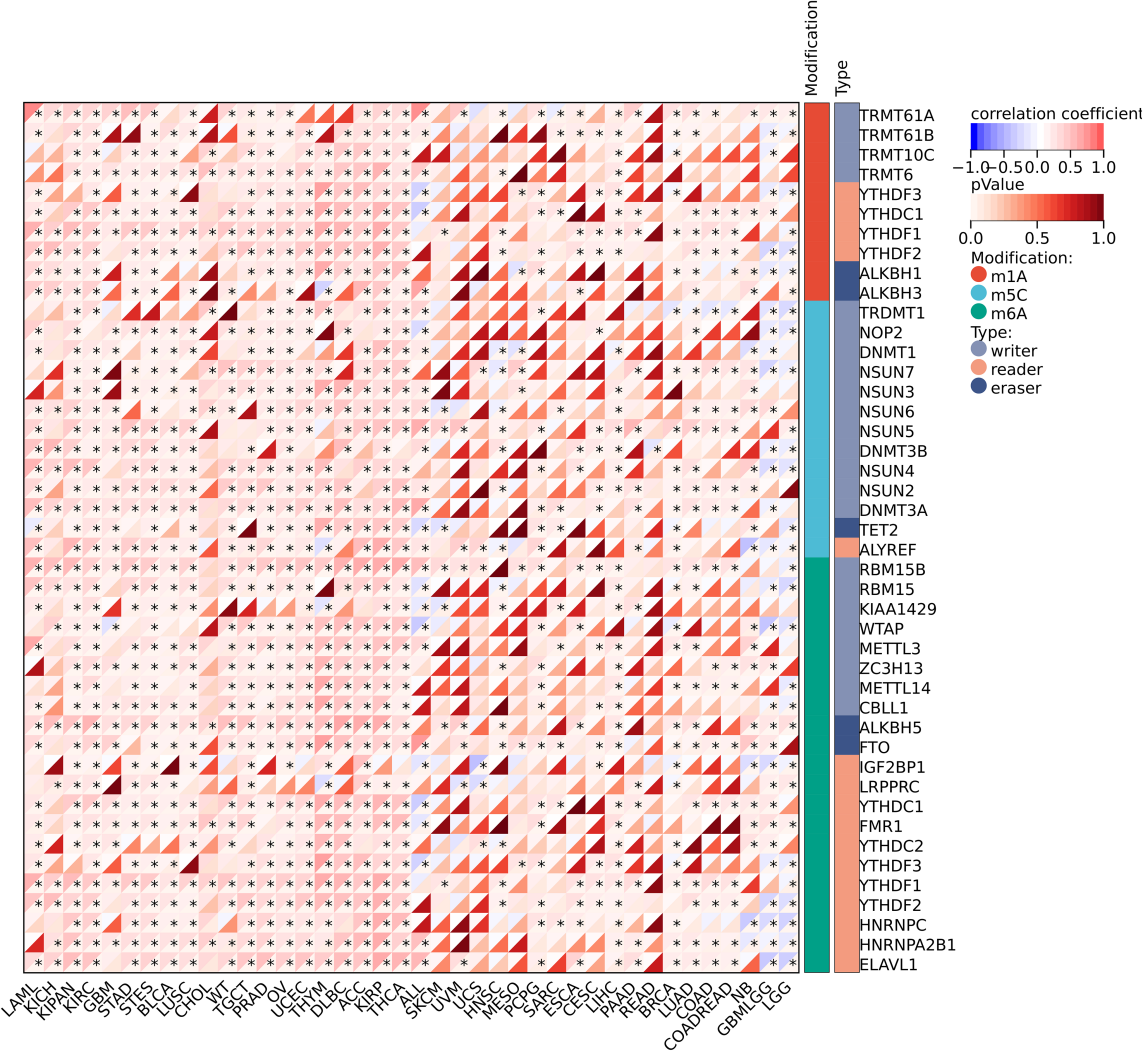
Pan-cancer landscape of IGSF8 association with RNA methylation regulators. *p < 0.05.

We further investigated the correlation between the expression level of IGSF8 and tumor heterogeneity and stemness (**Figures 5A–D**). In the term of TMB, we observed a significant correlation in 12 types of tumors, with 7 showing a significant positive correlation and 5 showing a significant negative correlation (**Figure 5A**). MSI and NEO are indicators of tumor response to immunotherapy. For MSI, we observed a significant correlation in 9 types of tumors, with 8 showing a significant positive correlation and 1 showing a significant negative correlation (**Figure 5B**). However, NEO was only correlated with IGSF8 expression in KICH (r=-0.50) (**Figure 5C**). Tumor purity refers to the proportion of tumor cells in a sample compared to non-tumor cells. High tumor purity means the sample is predominantly tumor cells, while low purity indicates a significant presence of normal or other non-tumor cells. We observed a significant correlation between IGSF8 expression and tumor purity in 25 types of tumors, with 23 showing a significant positive correlation and 2 showing a significant negative correlation (**Figure 5D**). In the term of RNAss, IGSF8 exhibited prominent correlations with 20 types of tumors, with 7 showing a significant positive correlation and 13 showing a significant negative correlation (**Figure 5E**). For DNAss, IGSF8 exhibited prominent correlations with 10 types of tumors, with 7 showing a significant positive correlation and 3 showing a significant negative correlation (**Figure 5F**). Next, we analyzed the IGSF8 mutation status in pan-cancer. IGSF8 alteration was observed in 22 cancers, with a mutation frequency of 4.0% for UCEC, 2.7% for DLBC, 2.2% for LUAD and 2.1% for COAD (**Figure 6A**). In five different types of cancer, we divided patients into high-expression and low-expression groups based on the median expression level of IGSF8 and compared the differences in the mutation landscape between the two expression groups (**Figures 6B–F**). In COAD, top 5 significant gene mutations including APC, MUC16, PIK3CA, DNAH5 and MUC5B were observed between high- and low- IGSF8 expression groups (**Figure 6B**). In LGG, top 5 significant gene mutations including TP53, ATRX, CIC, FUBP1 and RIMBP2 were observed between high- and low- IGSF8 expression groups (**Figure 6C**). Likewise, LIHC showed significant gene mutations, such as CTNNB1, HMCN1, DNAH7, FN1 and DCDC1 between the two groups (**Figure 6D**). LUSC showed significant gene mutations including ZNF804B, ZNF804A, EPHA5, NCAM2 and FSHR between the two groups (**Figure 6E**). STAD showed significant gene mutations including TP53, LAMA1, DNAH11, APOB and PREX2 between the two groups (**Figure 6F**). In terms of RNA methylation, we found that IGSF8 expression was closely associated with multiple m1A, m5C and m6A modification in KIRC, KIRP, ACC and thymoma (THYM) (**Figure 7**).

### Cell proliferation and colony formation

**Figure 8:**
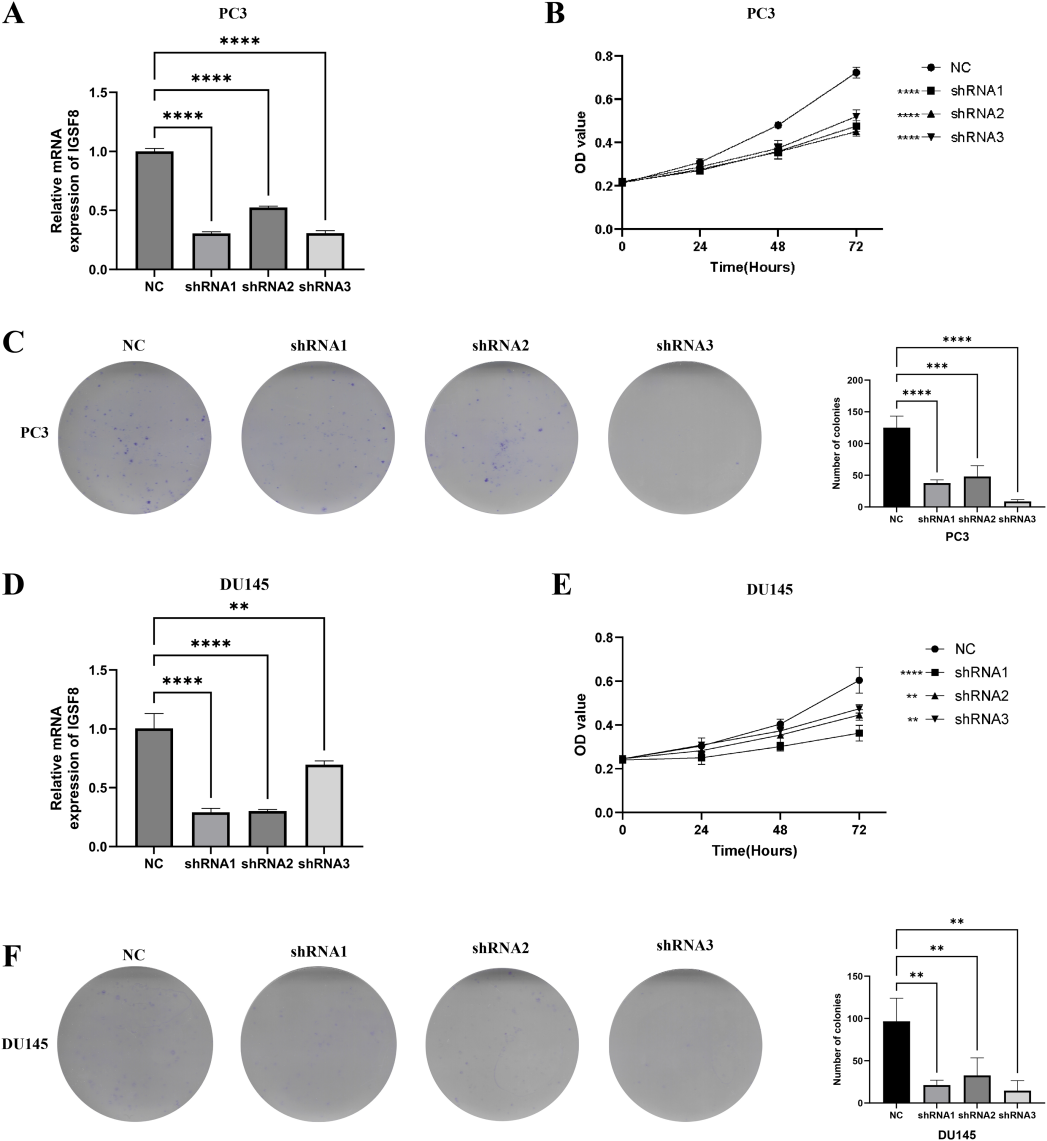
IGSF8 mediates prostate cancer cell proliferation. **(A)** IGSF8 mRNA expression was detected in PC3 cells with IGSF8 knockdown; **(B)** effect of IGSF8 knockdown on PC3 cells using CCK8 assay; **(C)** effect of IGSF8 knockdown on PC3 cells using clone formation assay; **(D)** IGSF8 mRNA expression was detected in DU145 cells with IGSF8 knockdown; **(E)** effect of IGSF8 knockdown on DU145 cells using CCK8 assay; **(F)** effect of IGSF8 knockdown on DU145 cells using clone formation assay.

We found that IGSF8 expression was downregulated in prostate cancer cells after transfection of the three shRNAs using the RT-qPCR assay (**Figures 8A, D**). The CCK-8 cell proliferation assay results showed that IGSF8 knockdown significantly impaired the proliferation ability of PC3 and DU145 cells (**Figures 8B, E**). Moreover, the colony formation assay showed that the colony formation ability of PC3 and DU145 cells was significantly inhibited after IGSF8 knockdown (**Figures 8C, F**).

## Discussion

IGSF8, also known as EWI-2 or CD316, is a transmembrane protein that belongs to the immunoglobulin superfamily (IgSF). It has been widely studied in various physiological contexts for its role in cell adhesion, migration, and signal transduction through interactions with tetraspanins like CD9 and CD81 (13). In non-oncological research, IGSF8 is crucial in neural development, particularly in organizing synaptic connections within the hippocampal CA3 microcircuit, which is vital for cognitive processes and neural plasticity (38). IGSF8 also plays a significant role in immune regulation, where it modulates immune responses. Although it has been suggested to be dispensable for fertility in mouse models, this might indicate a tissue-specific or condition-specific role (39). Additionally, IGSF8’s involvement in olfactory sensory neuron development further highlights its diverse functional roles across different biological systems (16).

In the context of oncology, IGSF8 has gained attention due to its oncogenic potential across various cancer types. It has been identified as a key regulator in maintaining the stemness of myeloid leukemia cells by inhibiting the degradation of β-catenin, thus promoting leukemogenesis and therapy resistance (12). In gliomas, IGSF8 has been recognized as a potential therapeutic target due to its role in enhancing tumor cell invasiveness and resistance to conventional treatments, making it a critical factor in glioma progression (18). Furthermore, studies have shown that IGSF8 may contribute to the progression of melanoma by negatively regulating TGF-β signaling, further demonstrating its involvement in tumor growth and metastasis (40). The current study builds upon these findings by conducting a pan-cancer analysis of IGSF8, revealing its broader oncogenic role across multiple cancer types. Unlike previous research, which has primarily focused on specific cancers, our study provides a comprehensive overview of IGSF8’s dual role in promoting tumor growth and modulating immune responses, suggesting its potential as a universal therapeutic target in cancer (23). This expanded understanding of IGSF8’s function underscores the necessity for further exploration of its role as a critical mediator in tumor biology and immune evasion. Although research in the field of cancer is limited, some studies have indicated that IGSF8 expression may serve as a biomarker for ovarian cancer (41). Additionally, *in vitro* experiments have demonstrated that IGSF8 expression is associated with the growth of androgen-deficient prostate cancer cells (42). Our study revealed that IGSF8 exhibited differential expression in various tumors, including UCEC, BRCA, LUAD, PRAD, and STAD, suggesting its relevance to solid tumors. Furthermore, we observed a correlation between IGSF8 expression and advanced age across multiple tumor types. Aging is a dynamic process, and the accompanying epigenetic changes contribute to tumor occurrence and development (43–46). The association between IGSF8 expression in tumors and advanced age underscores the importance of investigating the genetic interplay between aging and tumorigenesis.

This study investigated the correlation between IGSF8 expression levels and immune regulatory genes, immune checkpoints, and tumor-infiltrating cells. Our results consistently demonstrate a strong and recurrent positive correlation between IGSF8 and CD276 (also known as B7-H3) across multiple solid tumors. CD276 is a transmembrane immune checkpoint molecule expressed on various tumor and stromal cells, exerting context-dependent co-stimulatory or co-inhibitory effects (47, 48). It is well recognized for its role in facilitating tumor immune evasion by suppressing T cell activation and reducing cytokine secretion (49, 50), and its high expression is associated with poor prognosis in numerous cancer types (51). Currently, CD276-targeted monoclonal antibodies and antibody-drug conjugates are undergoing clinical trials as promising cancer immunotherapies (52). Beyond co-expression, our findings raise the possibility of functional synergy between IGSF8 and CD276. IGSF8 is known to cluster in TEMDs by interacting with CD9 and CD81, which are involved in the trafficking and membrane stability of transmembrane proteins. It is plausible that IGSF8 may stabilize or facilitate the surface retention of CD276 within these microdomains, thereby amplifying its immunosuppressive effects in the TME. The co-localization and cooperative immunomodulatory functions of these two molecules may constitute a dual mechanism driving immune escape, especially in immunologically “cold” tumors. From a therapeutic standpoint, co-targeting IGSF8 and CD276 may provide synergistic benefits by simultaneously enhancing T and NK cell activity and reversing tumor immune evasion. Future functional studies are warranted to investigate whether IGSF8 directly regulates CD276 expression, localization, or recycling, and to assess the immune and clinical consequences of their co-inhibition.

The TME plays a central role in regulating cancer progression, therapeutic response, and immune evasion (53, 54). It consists of a dynamic interplay between malignant cells, immune infiltrates, stromal elements and extracellular matrix components, which collectively determine the balance between tumor-promoting and tumor-suppressive forces (55, 56). In this context, IGSF8 has emerged as a key modulator of immune cell behavior. Li et al. (23) demonstrated that IGSF8 acts as an innate immune checkpoint by interacting with NK cell inhibitory receptors, thereby suppressing NK-mediated cytotoxicity. Their study further showed that blockade of IGSF8 restores NK function and enhances antigen presentation and T cell activation *in vivo*, underscoring its mechanistic role in immune escape. While Li et al. (23) uncovered a direct molecular mechanism in selected tumor models, our pan-cancer analysis expands this paradigm by demonstrating that IGSF8 expression is broadly correlated with alterations in immune cell infiltration across multiple solid tumor types. In addition, IGSF8 expression was associated with shifts in stromal components such as cancer-associated fibroblasts and macrophages, further indicating its influence on both structural and immunological features of the TME. These findings position IGSF8 not only as a molecular immune checkpoint but also as a regulatory hub in tumor ecosystems, supporting its therapeutic relevance in diverse oncologic contexts.

The analysis of the mutation landscape across different cancer types, based on IGSF8 expression levels, shows distinct patterns of gene mutations. This suggests that IGSF8 may be involved in regulating specific oncogenic pathways in different cancers. In COAD, mutations in APC and PIK3CA are well-known drivers of tumorigenesis. APC is central to the Wnt signaling pathway, and its mutation leads to uncontrolled cell proliferation, while PIK3CA mutations activate the PI3K-Akt pathway, which promotes tumor cell survival and growth (57). The correlation between IGSF8 expression and these mutations suggests that IGSF8 may modulate these critical pathways, potentially affecting tumor proliferation or response to targeted therapies. In LGG, TP53 and ATRX mutations are frequent in gliomas. TP53 is a tumor suppressor that controls the cell cycle and apoptosis, and its mutation disrupts genomic stability (58). ATRX mutations impact chromatin remodeling and telomere maintenance (59). IGSF8’s association with these mutations hints at a role in DNA repair mechanisms or in controlling glioma cell differentiation and survival, particularly in the context of these chromatin and genome stability regulators. Notably, in LIHC, mutations in CTNNB1, which encodes β-catenin, drive aberrant activation of the Wnt/β-catenin signaling pathway, contributing to liver cancer progression (60). The connection between IGSF8 and CTNNB1 mutations in liver cancer suggests that IGSF8 could influence the regulation of Wnt signaling, possibly affecting tumor cell growth or differentiation. Overall, the mutation landscape analysis suggests that IGSF8 might act as a modulator of oncogenic pathways, influencing tumor progression and potentially serving as a biomarker for identifying mutation-driven therapeutic targets.

Importantly, although our study identifies BX-795 and tozasertib as candidate compounds for tumors with high IGSF8 expression, neither drug has yet been approved for clinical use in oncology (61, 62). To validate their therapeutic relevance, future studies should evaluate their efficacy across cancer cell line panels, organoid model and patient-derived xenograft (PDX) models (63). BX-795, a TBK1 and IKKϵ inhibitor (64), may be tested alone or in combination with immunomodulatory genes to determine whether it can enhance immune cell activity and suppress immune evasion in tumors characterized by IGSF8 and CD276 co-expression. These approaches may uncover synergistic anti-tumor effects that support clinical translation. PDX models preserve patient-specific tumor architecture, histological features, and microenvironmental interactions more effectively than conventional xenografts. Therefore, they represent a more reliable system for preclinical drug evaluation. Several recent studies have highlighted the power of PDX models in translational oncology research. For example, they have been used to investigate the effects of anesthetic techniques on breast cancer metastasis, to dissect how metabolic pathways contribute to colorectal cancer development, and to validate the *in vivo* functions of tumor-suppressive microRNAs in lung cancer (65–67). Incorporating PDX models, potentially augmented by humanized immune systems, would enhance the biological and translational relevance of future IGSF8-targeted drug studies. Such approaches may accelerate the development of precision therapies for immunologically cold tumors where IGSF8 acts as a central immune regulatory hub.

While this work offers a broad investigation into the biological and clinical relevance of IGSF8, several limitations should be acknowledged. First, although preliminary *in vitro* experiments confirmed the biological effects of IGSF8, further *in vivo* validation is essential to clarify its mechanistic role in tumor progression and therapeutic modulation. Second, the analyses rely on publicly available datasets such as TCGA, which are susceptible to cohort selection bias, batch effects, and incomplete clinical annotation (68). These factors may affect the accuracy and generalizability of the conclusions, particularly in the context of patient heterogeneity. In addition, the immune microenvironment analysis employed the EPIC deconvolution algorithm, which is not considered a gold standard. Bulk transcriptomic approaches may misestimate immune cell proportions and fail to detect rare or spatially distinct populations (69). To improve resolution and accuracy, future studies should incorporate multiplex immunohistochemistry (mIHC) for spatial mapping of immune and stromal components, and single-cell RNA sequencing (scRNA-seq) to characterize cell-specific expression and validate deconvolution results (70–72). These complementary methods would provide more refined insight into the immunological role of IGSF8. Despite these limitations, the present findings offer a solid framework for further exploration. Incorporating multi-modal and high-resolution technologies in subsequent research will help to strengthen mechanistic understanding and accelerate translational development of IGSF8 as a potential therapeutic target.

## Conclusions

In summary, this study provides a comprehensive characterization of IGSF8 across multiple cancer types, integrating transcriptomic data analysis with preliminary *in vitro* validation. We demonstrate that IGSF8 expression is associated with unfavorable prognosis, enhanced tumor cell proliferation, and suppression of immune infiltration in several malignancies. Importantly, these findings highlight IGSF8’s dual role as both an oncogenic driver and an immune regulator, positioning it as a central modulator of tumor progression and immune evasion. This dual functionality underscores the potential of IGSF8 as a candidate for prognostic stratification and targeted therapy development. Future research should prioritize mechanistic studies and translational efforts to explore IGSF8-directed interventions in immunologically cold tumors.

## Data Availability

The original contributions presented in the study are included in the article/**Supplementary Material**. Further inquiries can be directed to the corresponding authors.

## Supplementary material

Supplementary Figure 1 The correlation between IGSF8 expression and clinical features. **(A)** the correlation between IGSF8 expression and T stages at pan-cancer level; **(B)** the correlation between IGSF8 expression and N stages at pan-cancer level; **(C)** the correlation between IGSF8 expression and M stages at pan-cancer level; **(D)** the correlation between IGSF8 expression and grades at pan-cancer level; **(E)** the correlation between IGSF8 expression and clinical stages at pan-cancer level; **(F)** the differential expression of IGSF8 between female and male at pan-cancer level; **(G)** the correlation between IGSF8 expression and ages at pan-cancer level.

Supplementary Figure 2 The correlation between IGSF8 expression and tumor-infiltrating cells using CIBERSORT, MCPcounter, Timer and xCELL algorithm. **(A)** the correlation between tumor-infiltrating cells and IGSF8 at pan-cancer level using the CIBERSORT algorithm; **(B)** the correlation between tumor-infiltrating cells and IGSF8 at pan-cancer level using the MCPcounter algorithm; **(C)** the correlation between tumor-infiltrating cells and IGSF8 at pan-cancer level using the Timer algorithm; **(D)** the correlation between tumor-infiltrating cells and IGSF8 at pan-cancer level using the xCELL algorithm.

## Glossary

IGSF8: Immunoglobulin superfamily member 8
TEMDs: tetraspanin-enriched membrane domains
TME: tumor microenvironment
TCGA: The Cancer Genome Atlas
OS: overall survival
DSS: disease-specific survival
DFS: disease-free survival
PFI: progression-free interval
TMB: tumor mutation burden
NEO: neoantigens
MSI: microsatellite instability
DNAss: DNA methylation score
RNAss: RNA expression score
COAD: Colon adenocarcinoma
LGG: Brain Lower Grade Glioma
LIHC: Liver hepatocellular carcinoma
LUSC: Lung squamous cell carcinoma
STAD: Stomach adenocarcinoma
GDSC: Genomics of Drug Sensitivity in Cancer
CTRP: Cancer Treatment Response Portal
RT-qPCR: Real-time quantitative polymerase chain reaction
qPCR: quantitative PCR
OD: optical density
UCEC: Uterine Corpus Endometrial Carcinoma
BRCA: Breast invasive carcinoma
LUAD: Lung adenocarcinoma
ESCA: Esophagus carcinoma
STES: Stomach and Esophageal carcinoma
COADREAD: Colon adenocarcinoma/Rectum adenocarcinoma
PRAD: Prostate adenocarcinoma
HNSC: Head and Neck squamous cell carcinoma
SKCM: Skin Cutaneous Melanoma
BLCA: Bladder Urothelial Carcinoma
THCA: Thyroid carcinoma
OV: Ovarian serous cystadenocarcinoma
PAAD: Pancreatic adenocarcinoma
UCS: Uterine Carcinosarcoma
ALL: Acute Lymphoblastic Leukemia
LAML: Acute Myeloid Leukemia
PCPG: Pheochromocytoma and Paraganglioma
ACC: Adrenocortical carcinoma
CHOL: Cholangiocarcinoma
CESC: Cervical squamous cell carcinoma and endocervical adenocarcinoma
KIPAN: Pan-kidney cohort
GBMLGG: Glioma
CAFs: cancer associated fibroblasts
THYM: thymoma
IgSF: immunoglobulin superfamily
PDX: patient-derived xenograft
mIHC: multiplex immunohistochemistry
scRNA-seq: single-cell RNA sequencing.
