# Supplementary figures and images for: Functional characterization and clinical significance of IGSF8 in pan-cancer: an integrated bioinformatic and experimental study
Source: Front Immunol. 2025 Aug 27;16:1642193. doi: 10.3389/fimmu.2025.1642193 (PMC12420624; doi:10.3389/fimmu.2025.1642193)

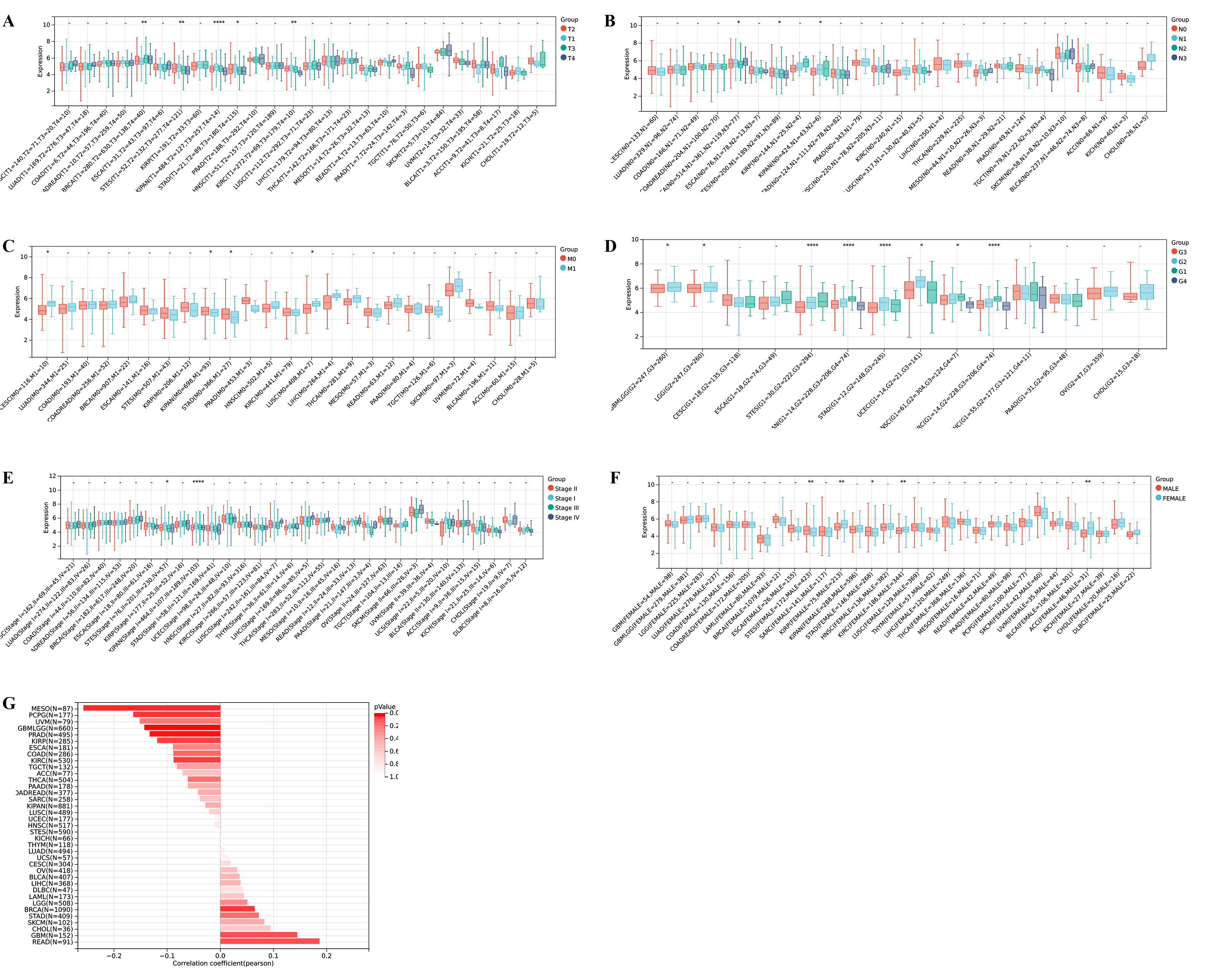

Supplement: Supplementary Figure 1 — The correlation between IGSF8 expression and clinical features. (A) the correlation between IGSF8 expression and T stages at pan-cancer level; (B) the correlation between IGSF8 expression and N stages at pan-cancer level; (C) the correlation between IGSF8 expression and M stages at pan-cancer level; (D) the correlation between IGSF8 expression and grades at pan-cancer level; (E) the correlation between IGSF8 expression and clinical stages at pan-cancer level; (F) the differential expression of IGSF8 between female and male at pan-cancer level; (G) the correlation between IGSF8 expression and ages at pan-cancer level. [file Image1.tif]

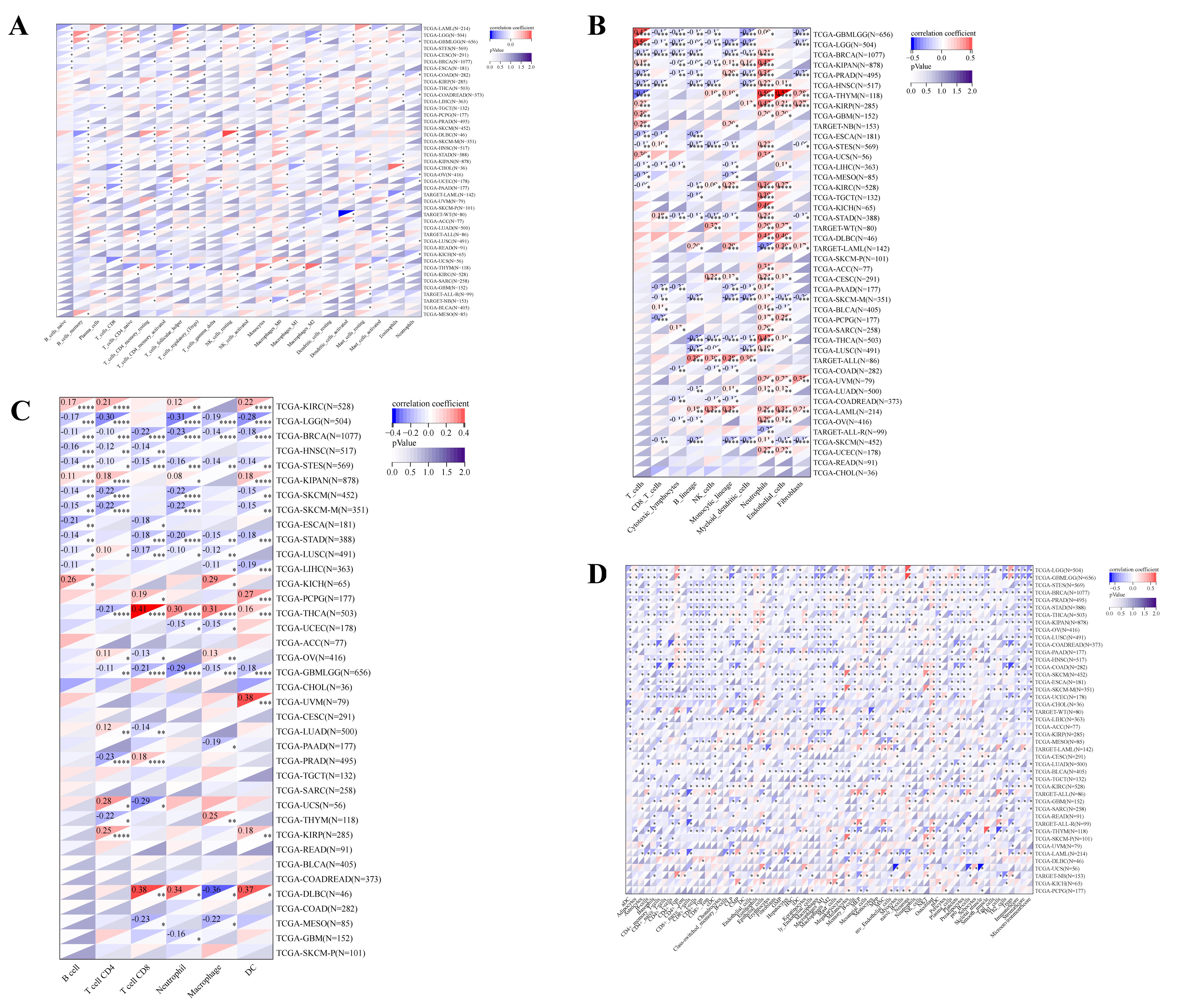

Supplement: Supplementary Figure 2 — The correlation between IGSF8 expression and tumor-infiltrating cells using CIBERSORT, MCPcounter, Timer and xCELL algorithm. (A) the correlation between tumor-infiltrating cells and IGSF8 at pan-cancer level using the CIBERSORT algorithm; (B) the correlation between tumor-infiltrating cells and IGSF8 at pan-cancer level using the MCPcounter algorithm; (C) the correlation between tumor-infiltrating cells and IGSF8 at pan-cancer level using the Timer algorithm; (D) the correlation between tumor-infiltrating cells and IGSF8 at pan-cancer level using the xCELL algorithm. [file Image2.tif]
